# Predicting the Severity and Prognosis of Trismus after Intensity-Modulated Radiation Therapy for Oral Cancer Patients by Magnetic Resonance Imaging

**DOI:** 10.1371/journal.pone.0092561

**Published:** 2014-03-21

**Authors:** Li-Chun Hsieh, John W. Chen, Li-Ying Wang, Yuk-Ming Tsang, Pei-Wei Shueng, Li-Jen Liao, Wu-Chia Lo, Yu-Chin Lin, Chien-Fu Tseng, Ying-Shiung Kuo, Jie-Yang Jhuang, Hui-Ju Tien, Hsueh-Fen Juan, Chen-Hsi Hsieh

**Affiliations:** 1 Division of Medical Imaging, Department of Radiology, Far Eastern Memorial Hospital, Taipei, Taiwan; 2 Division of Radiation Oncology, Department of Radiology, Far Eastern Memorial Hospital, Taipei, Taiwan; 3 Department of Otolaryngology, Far Eastern Memorial Hospital, Taipei, Taiwan; 4 Div. Medical Oncology and Hematology, Department of Internal Medicine, Far Eastern Memorial Hospital, Taipei, Taiwan; 5 Department of Dentistry and Oral Surgery, Far Eastern Memorial Hospital, Taipei, Taiwan; 6 Department of Anatomical Pathology, Far Eastern Memorial Hospital, Taipei, Taiwan; 7 Department of Medicine, School of Medicine, National Yang-Ming University, Taipei, Taiwan; 8 Institute of Traditional Medicine, School of Medicine, National Yang-Ming University, Taipei, Taiwan; 9 School and Graduate Institute of Physical Therapy, College of Medicine, National Taiwan University, Taipei, Taiwan; 10 Department of Radiation Oncology, National Defense Medical Center, Taipei, Taiwan; 11 Center for Systems Biology and Department of Radiology, Massachusetts General Hospital, Harvard Medical School, Massachusetts, United States of America; 12 Department of Life Science, Institute of Molecular and Cellular Biology, Center for Systems Biology, National Taiwan University, Taipei, Taiwan; 13 Graduate Institute of Biomedical Electronics and Bioinformatics, National Taiwan University, Taipei, Taiwan; 14 Medical Imaging Center, Taipei Medical University Hospital, Taipei, Taiwan; University of Washington School of Medicine, United States of America

## Abstract

To develop magnetic resonance imaging (MRI) indicators to predict trismus outcome for post-operative oral cavity cancer patients who received adjuvant intensity-modulated radiation therapy (IMRT), 22 patients with oral cancer treated with IMRT were studied over a two-year period. Signal abnormality scores (SA scores) were computed from Likert-type ratings of the abnormalities of nine masticator structures and compared with the Mann-Whitney U-test and Kruskal–Wallis one-way ANOVA test between groups. Seventeen patients (77.3%) experienced different degrees of trismus during the two-year follow-up period. The SA score correlated with the trismus grade (r = 0.52, p<0.005). Patients having progressive trismus had higher mean doses of radiation to multiple structures, including the masticator and lateral pterygoid muscles, and the parotid gland (p<0.05). In addition, this group also had higher SA-masticator muscle dose product at 6 months and SA scores at 12 months (p<0.05). At the optimum cut-off points of 0.38 for the propensity score, the sensitivity was 100% and the specificity was 93% for predicting the prognosis of the trismus patients. The SA score, as determined using MRI, can reflect the radiation injury and correlate to trismus severity. Together with the radiation dose, it could serve as a useful biomarker to predict the outcome and guide the management of trismus following radiation therapy.

## Introduction

Trismus is one of the sequela for head and neck cancer patients. The prevalence of trismus after head and neck oncology treatment could be as high as 42% [1]. It has been described as any type of restriction in the opening of the mouth including radiation and conditions after trauma, surgery, or tetanus [2], [3], [4]. Radiation therapy involving the temporomandibular joint (TMJ), pterygoid muscles, and the temporalis or the masseter muscle is most likely to result in trismus [5], [6]. Moreover, there may be scar tissue from radiation, surgery, nerve damage, or a combination of these factors to cause trismus [6], [7]. Further, doses of radiotherapy (RT) in excess of 60 Gy [8] or the configuration of the radiation field increasing [9] are more likely to cause trismus.

Recently, extensive data suggest intensity-modulated radiation therapy (IMRT) is safe and efficacious in the adjuvant setting for oral cavity cancer (OCC) [10], [11], [12]. Hsiung et al. [13] and Chen et al. [14] noted radiation induced trismus for nasopharyngeal carcinoma patients progressed over time and improved by IMRT. Louise et al. [15] noted when doses of massetor or pterygoid muscles larger than 55 Gy were given, the incidences of trismus for head and neck patients were as high as 45%. With every additional 10 Gy to the pterygoid muscle, the increase in the probability of trismus was 24% [5].

Magnetic resonance imaging (MRI) can display abnormal image findings in masticator structures for patients with trismus developing after radiotherapy for nasopharyngeal carcinoma (NPC) [6]. However, in this report, the image findings showed no correlation between the severity of trismus and radiation dosage [6]. Moreover, aggressive interventions should be given to the patient whose trismus does not improve as time passes. However, there is still no good indicator to predict the outcome of trismus developing after radiotherapy.

The purpose of this study is to use MRI to predict the severity of trismus and evaluate the correlation of images and radiation dosage between related structures concerning trismus. We also tried to find the prognostic factors of trismus developing after the operation and IMRT for oral cancer patients.

## Materials and Methods

### Patient characteristics

This retrospective study was approved by the Institutional Review Board (IRB) of the Far Eastern Memorial Hospital (FEMH-IRB-101127-F) and Clinical Trials government with number of NCT02004639. Patient consents were specifically waived because the data were analyzed anonymously and approval was given by the IRB. Patient confidentiality and privacy were protected according to national standards. Between December 2006 and December 2012, patients with OCC squamous cell carcinoma (SCC) who had undergone surgery followed by postoperative IMRT with or without chemotherapy at Far Eastern Memorial Hospital were enrolled. Patients who were treated for recurrent SCC of the oral cavity (including neck recurrences), follow-up time less than 2 years, incomplete trismus grading records or incomplete MRI image studies were excluded from the analysis. The disease was staged according to the American Joint Committee on Cancer Staging Classifications 6^th^ edition, which was based on the pathological findings after radical surgery.

### Radiation therapy

**Figure 1:**
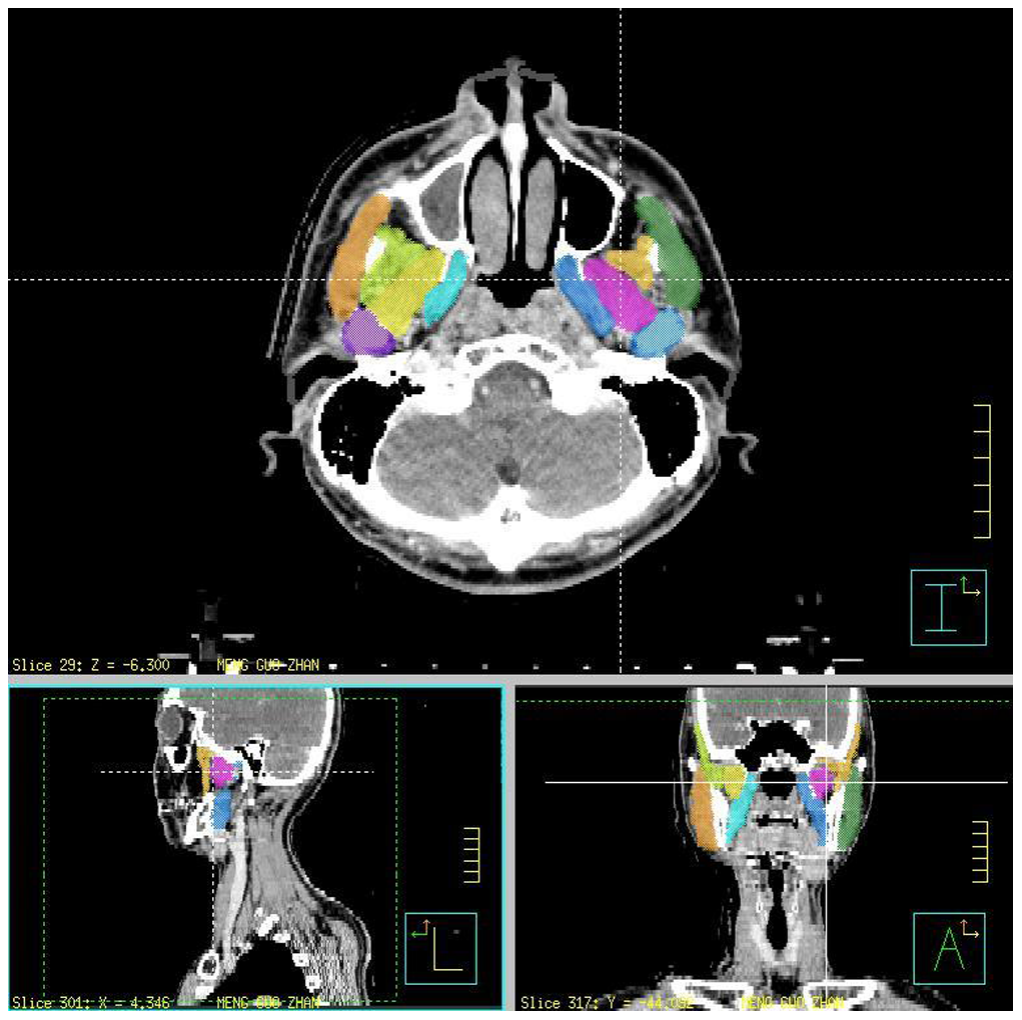
Describe the trismus related muscles and structures that were targeted by colors in one of enrolled patients. Light blue: left side medial pterygoid muscle; Pink: left side lateral pterygoid muscle; Light orange: left side temporalis; Forest green: left side masseter; Steel blue: left side temporomandibular joints. Sky blue: right side medial pterygoid muscle; Yellow: right side lateral pterygoid muscle; Yellow green: right side temporalis; Orange: right side masseter; Purple: right side temporomandibular joints.

A 7-filed IMRT or helical tomotherapy, image-guided IMRT, with daily fractions of 1.8 or 2 Gy in five consecutive days were used. It encompassed the preoperative gross tumor and postoperative flap plus a 0.8- to 1-cm margin, including the resection bed with soft-tissue invasion by the tumor or extra-capsular extension (ECE) that received 60–66 Gy in 30–33 fractions; 64–66 Gy was delivered to high-risk OCC patients and 60 Gy was delivered to intermediate-risk OCC patients. For the high-risk subclinical area, 59.4–60 Gy/30–33 fractions were delivered and for the low-risk area of potential subclinical disease, 51.2–54 Gy/30–33 fractions were delivered [16]. The grading of trismus in the clinical results was from grade one to three according to the Common Terminology Criteria for Adverse Events (CTCAE) v3.0. The medial and lateral pterygoid, masseter and temporalis muscles, parotid gland, mandibular rami and temporomandibular joints (TMJ) were targeted and calculated by dose-volume histogram for those enrolled patients retrospectively (Fig. 1).

### Evaluation of MRI examinations

MRI results at 6^th^ months, 12^th^ months and 24^th^ months after initiation of radiation treatment were selected for analysis. These data were assessed by two radiologists blind to their clinical information and trismus severity. The MRI imaging protocols applied at our institution for patients with oral cancer include axial T1 weighted image (T1W), coronal T2 weighted image (T2W), axial T2W with fat saturation, sagittal, coronal T1W post-gadolinium and axial T1W post-gadolinium with fat saturation sequences. TIW and T2W are both conventional image sequences applied in daily MRI examination. In practice, T1W and T2W image are where the contrast depends predominantly on the differences in the T1 times and T2 times between tissues e.g. fat and water, respectively [17]. Tl describes the interaction of excited nuclei with the surroundings (spin-lattice relaxation), while T2 describes the interaction of excited nuclear spins with the spins of other nuclei (spin-spin relaxation) [18].

Signal abnormality scores (SA score) were rated with a Likert scoring system [19]. A three-point Likert item SA score was used in the current study, and the structures analyzed were listed in table 1. For evaluation of the masticator muscles, we rated the signal change as “0” =  normal; “1” = T2 signal change only; “2” = 1 plus muscle atrophy or obvious abnormal enhancement on T1W post-gadolinium sequences. While rating the perimasticator space, “0” =  normal; “1” = T2 signal change only, “2” = 1 plus fibrosis tissue formation or abnormal enhancement. For chronic injury of the mandibular division of the trigeminal nerve, which was inferred if there was atrophy of all ipsilateral masticator muscles as well as of the mylohyoid and anterior belly of the digastric muscles, we rated atrophy of part of these muscles as “1” and complete atrophy of all muscles as “2”. In the parotid gland, only signal change was rated as “1” and obvious gland atrophy was rated as“2”. These abnormalities were differentiated from residual tumor by their signal characteristics and static appearance on serial imaging.

### Method of analysis

At first, groups according to trismus grade in CTCAE v3.0 were separated and compared using Kruskal–Wallis one-way ANOVA test without assuming parametric distribution. The correlation between the SA score and trismus grade, or the SA score and the radiation dose of individual masticator structures were tested by Spearman's correlation test. Statistically significant SA scores were used as independent variables, and determined as useful in predicting the severity of trismus. Using the SA scores found to be useful, the function of logistic regression was calculated and the receiver-operating characteristic (ROC) curve was generated to find an optimum cut-off for predicting trismus severity.

After that, the patients were re-separated into two groups according to their trismus condition. One was the “good prognosis, GP” group if patient's trismus grade improved in time sequence or as normal. The other was “poor prognosis, PP” group if patient's trismus remained stable or got worsened during the two year follow up. The SA scores at 6^th^ months, 12^th^ months, the radiation dose of all and individual masticator structures, along with the product of the SA score with mean radiation dose of masticator muscles (SA score × mean radiation dose of masticator muscles for specific interesting) were all compared between these two groups. All data were evaluated with the non-parametric Mann-Whitney U-test without assuming parametric distribution.

The statistically significant variables were used as independent factors, and only the ones showing statistical significance were determined as useful in predicting the incidence of poor prognosis trismus. Utilizing the function of logistic regression, propensity score was sought for estimating the incidence of poor prognosis trismus among all the subjects during the follow-up period [18].

All the above statistical analyses were performed using Prism (release 6.0, GraphPad Software Inc. La Jolla, CA, USA) and SPSS (IBM Corp. Released 2012. IBM SPSS Statistics for Windows, Version 21.0. Armonk, NY) and a P value equal or less than 0.05 was considered statistically significant.

## Results

Totally, 87 patients were enrolled but sixty-five cases were excluded due to incomplete clinical information or incomplete imaging data. Twenty-two cases with a total of sixty-six MRI exams were enrolled in this study. Table 2 lists the characteristics of the patients. The mean age was 50 years old, consisting of twenty men and two women. The dominant subgroups were buccal cancer (54.5%) and tongue cancer (36.4%). Ninety percent were stage III and IV. The median radiation dose was 64 Gy. Seventeen patients (77.3%) experienced different degrees of trismus during two year follow up after IMRT.

**Table 1:** Signal Abnormality Score in different masticator structures.

Anatomy	SA Score		
	0	1	2
**Masticator Muscles**			
Medial pterygoid			
Lateral Pterygoid			
Masseter	Normal	T2 signal change	T2 signal change with abnormal enhancement
Temporalis			
Masticator atrophy	Normal	Part of masticator muscle atrophy	All muscles atrophy
**Temporomandibular Joint**			
Deformity	Normal	Mild	Severe
Ramus ORN	Normal	Signal change	Bone destruction
**Other Structures**			
Perimasticator space	Normal	Inflammation change	Fibrosis
Parotid gland	Normal	Abnormal enhancement	Atrophy

Abbreviation.

SA score  =  Signal Abnormality score; ORN  =  Osteoradionecrosis.

**Figure 2:**
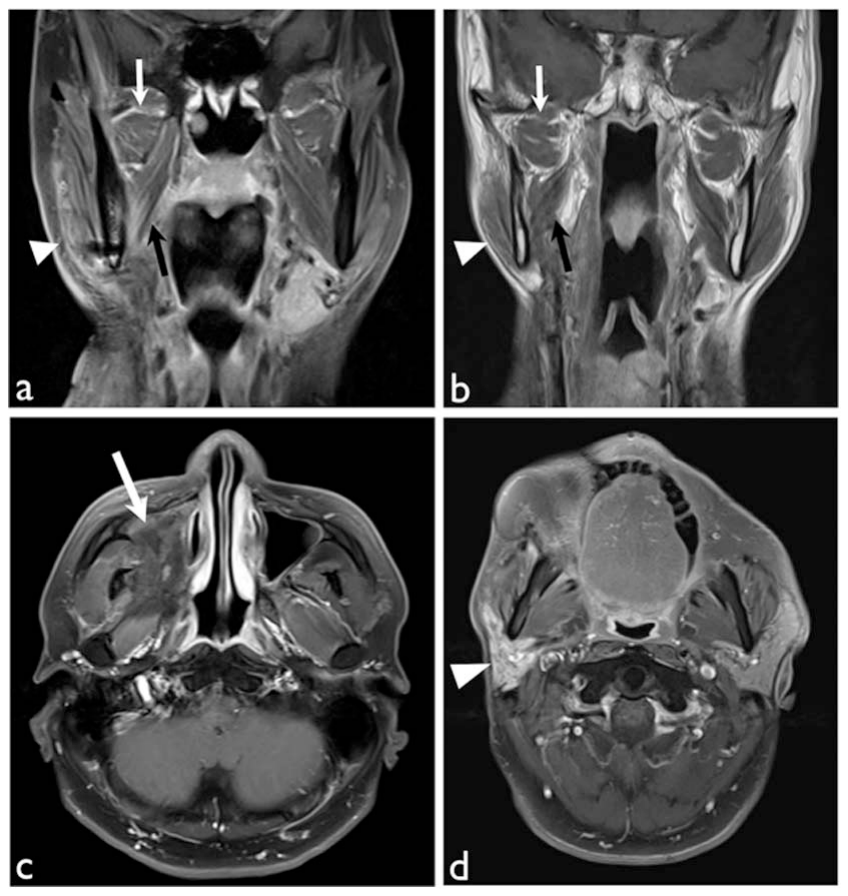
Illustrated T1W MRI post-contrast enhancement of trismus patient after RT. (a) Coronal view with fat saturation showed radiation effects in right lateral (white arrows), medial (black arrows) pterygoid muscles and masseter muscle (white arrowhead) with increased enhancement. (b) Coronal view showed atrophic change of the right lateral (white arrows), medial (black arrows) pterygoid muscles and masseter muscle (white arrowhead). (c) Axial view with fat saturation showed remarkable fibrotic tissue (white arrow) occupying right maxillary sinus and pterygoid space. (d) Axial view with fat saturation showed increased enhancement with atrophic change of the right parotid gland. All these findings were rated as point 2 according to our Likert scoring system of signal abnormality (SA score).

**Figure 3:**
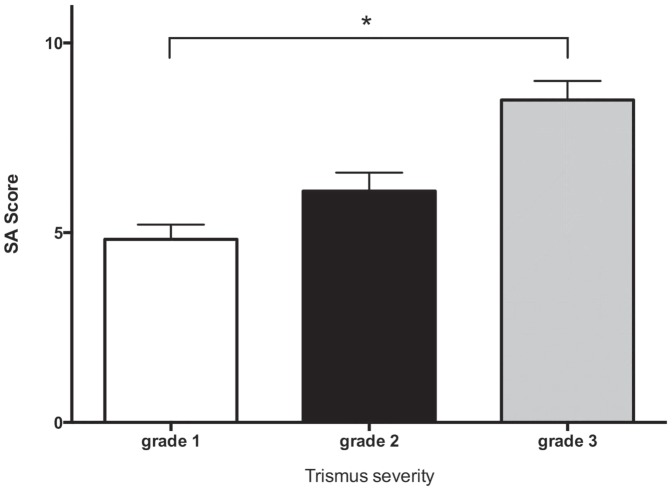
Comparison of signal abnormality scores between different groups of trismus severity. Significant difference in the SA scores between groups is noted (p = .03). The grade 3 group (most severe one) has the highest score and the grade 1 (least severe one) group has the lowest score.

Imaging analysis revealed all patients had various degrees of masticator muscle signal abnormality (Fig. 2a and 2b). Within them, denervation atrophy of the masticator muscles secondary to mandibular nerve damage (6/22, 9.0%), mild osteoradionecrosis change of mandibular rami (10/22, 15.2%), and perimasticator fibrosis extending into the masticator space totaled six (9.0%, Fig. 2c). Post RT damage of the parotid glands was 63.6% (Fig. 2d). The mean SA scores of the grade 1, 2 and 3 group are 4.87±1.9, 6.1±3.2 and 8.5±0.7, respectively. Significant differences between groups (Fig. 3, p = .03, one-way ANOVA test) were noted. Additionally, there was a significant positive correlation between SA score and trismus grading (r = 0.52, p<0.005) to predict the severity of trismus. In addition, the SA score of the trismus group (6.20±3.1, group of grade 2 and grade 3) was higher than that of the non-trismus group (4.87±1.9, grade 1 group, p = .04). The ROC curve was then generated using the values of the SA scores from the non-trismus (grade 1) and trismus (grade 2 plus grade 3) groups. The best cut-off values of the SA score that maximizes (sensitivity + specificity) were 5.5, with 64% sensitivity and 61% specificity.

**Figure 4:**
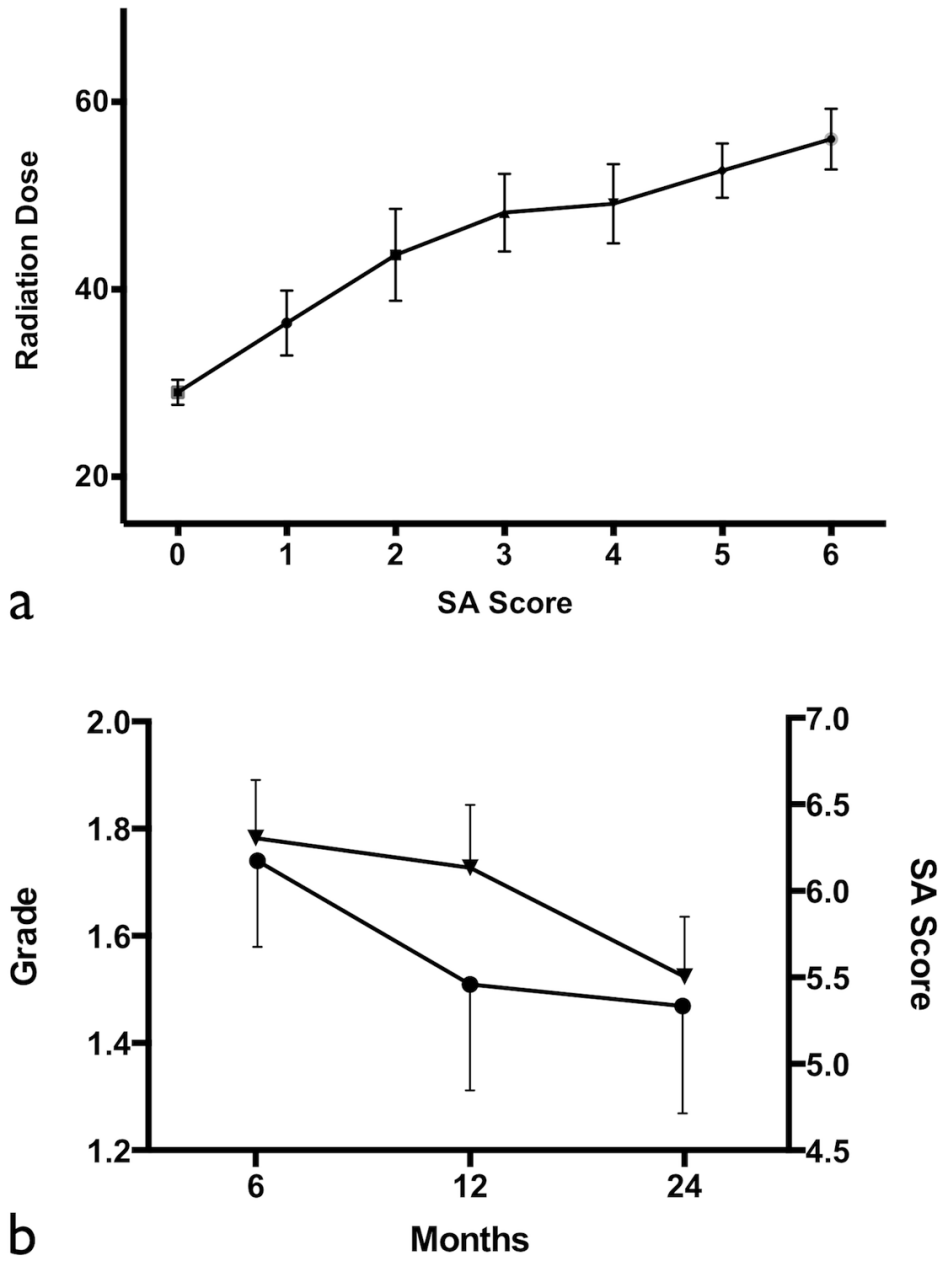
Correlation of SA score with radiation dose and the time trend of trismus development. (a) Correlation of the received radiation dose of individual masticator structures with their two year SA scores summation (r = 0.48, p<0.0001). (b) Evolution of the SA scores and the trismus grade over time. Gradual decrease of trismus severity grade (▾) and SA scores (□) is noted within 24 months.

There was a positive correlation between the dose of the individual masticator structures and the two year SA scores summation (r = 0.48, p<0.0001; Fig. 4a). Furthermore, the SA score gradually decreased along with clinical trismus grade and time sequence (Fig. 4b).

**Figure 5:**
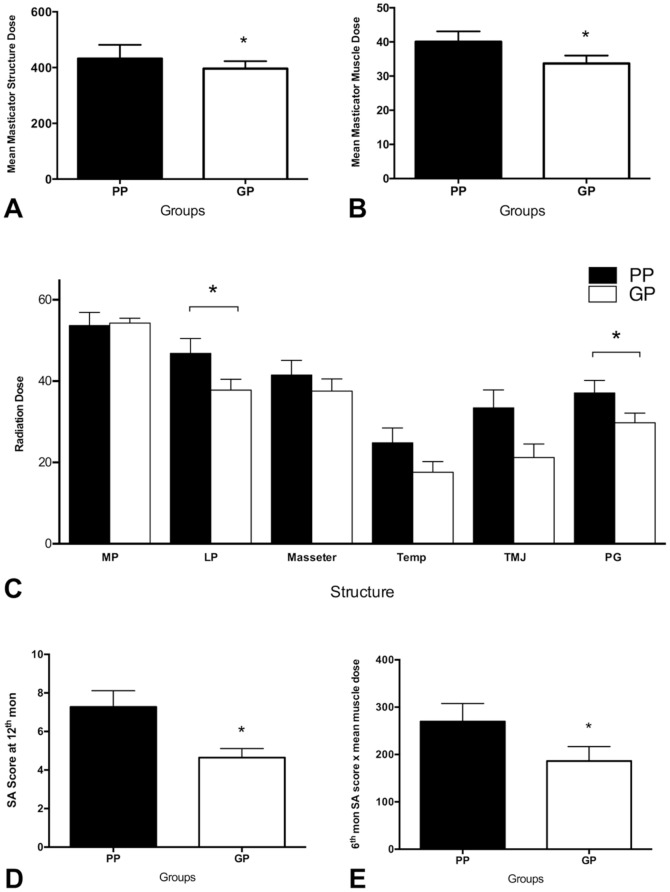
Comparison of radiation doses and SA scores between good prognosis and poor prognosis trismus groups. A significant difference is noted in the mean dose of masticator structures (p = .04; Fig. 5a), mean dose of masticator muscles (p = .05; Fig. 5b), mean dose of lateral pterygoid muscles (p = .04; Fig. 5c), mean dose of parotid glands (p = .01; Fig. 5c), the product of the six month SA score with mean masticator muscles dose (p = .04; Fig. 5d) and the twelve month SA score (p = .01; Fig. 5e). (MP: medial pterygoid muscle; LP: lateral pterygoid muscle; Temp: temporalis muscle; TMJ: temporomandibular joint; PG: parotid gland; SA score: signal abnormality score; GP: good prognosis; PP: poor prognosis).

There were significant differences between PP and GP groups in the mean dose of masticator structures (39.6±9.3 vs 33.3±7.1, p = .04; Fig. 5a), masticator muscles (40.0±10.1 vs 33.7±7.4, p = .05; Fig. 5b), lateral pterygoid muscle (46.8±17.3 vs 37.8±11.8, p = .04; Fig. 5c), parotid gland (37.1±14.3 vs 29.8±10.7, p = .01; Fig. 5c), and the product of six month SA scores with mean masticator muscle dose (269.6±126 vs 186.2±97.0, p = .04; Fig. 5d) and twelve month SA scores (7.3±2.8 vs 4.6±1.6, p = .01; Fig. 5e). There was no significant difference in six month SA scores, radiation dose of medial pterygoid muscle, masseter muscle, and the temporalis muscle between the PP and GP groups (Fig. 5c). However, there was a trend for the mean dose of TMJ to influence both groups (p = .06).

The best cut-off values of the above data that maximized sensitivity and specificity were obtained and listed in table 3. The functional formula for predicting the incidence of poor prognosis trismus, i.e. propensity score, came out as follows:

**Table 2:** Patient characteristics.

	Enrolled patients (No. = 22)
Variable	No. of patients (%)
Age (years)	
Mean	50
Range	38–71
Gender	
Male	20 (94.3%)
Female	2 (5.7%)
Subsite	
Oral tongue	8 (36.4%)
Buccal mucosa	12 (54.5%)
Lip	1 (4.5%)
Gum	1 (4.5%)
Pathology	
Squamous cell carcinoma	22 (100%)
Tumor stage	
Stage I	1 (4.5%)
Stage II	1 (4.5%)
Stage III	7 (31.8%)
Stage IVA	13 (59.1%)
Stage IVB	0
Primary tumor stage	
T1	2 (9.1%)
T2	5 (22.7%)
T3	7 (31.8%)
T4a	8 (36.4%)
T4b	0
Regional lymph node stage	
N0	9 (40.9%)
N1	4 (18.2%)
N2a	0
N2b	8 (36.4%)
N2c	1 (4.5%)
N3	0
Adjuvant concurrent chemotherapy	
Yes	20 (90.9%)
No	2 (9.1%)
RT dose	
Median (range)	64 Gy (59.8–70 Gy)
Mean dose of	
Right temporomandibular joint	24.8±16.9 (Gy)
Right masseter muscle	37.3±15.9 (Gy)
Right temporalis	19.8±14.0 (Gy)
Right medial pterygoid muscle	52.6±13.1 (Gy)
Right lateral pterygoid muscle	40.8±16.3 (Gy)
Right parotid gland	30.1±11.4 (Gy)
Left temporomandibular joint	28.3±21.6 (Gy)
Left masseter muscle	39.4±18.4 (Gy)
Left temporalis	21.2±16.9 (Gy)
Left medial pterygoid muscle	52.6±13.1 (Gy)
Left lateral pterygoid muscle	41.85±17.0 (Gy)
Left parotid gland	34.9±14.9 (Gy)

**Table 3:** The incidence of poor prognosis trismus predicts by cut-off values of masticator structures, time schedual SA score and propensity score.

Cutoff Values from ROC curve results			
Data Set	Cutoff Value	Sensitivity (%)	Specificity (%)
Mean Radiation Dose of masticator structures	42.14	63.64	90
Mean Radiation Dose of masticator muscles	42.54	63.64	90
Mean Radiation Dose of LP	42.03	72.73	70
Mean Radiation Dose of PG	27.99	80.95	60
6^th^ month SA score × mean masticator muscle dose	240.6	72.73	90
12^th^ month SA score	5.5	72.73	72.73
Propensity Score*	0.38	100	91

Abbreviation.

LP  =  lateral pterygoid muscle; PG  =  parotid gland; ROC curve  =  receiver operating characteristic curve.

*The propensity score was generated from the above six parameters with logistic regression analysis.

Propensity score  = 1/(1+1/exp (mean dose of masticator structures ×3.576+ mean dose of masticator muscles ×−3.284+ lateral pterygoid muscle dose × 0.138+ parotid gland dose ×−0.296+ the product of 6^th^ month SA scores with mean masticator muscle dose ×0.001+12^th^ month SA scores ×1.281−12.654)).

The area-under-curve (AUC) was 0.93 for propensity score and the optimum cut-off points were 0.38. By applying the cut-off value, we achieved 100% sensitivity and 93% specificity to predict the prognosis of these trismus patients (Table 3).

## Discussion

In the current study, there is a good correlation between the MRI SA score with trismus severity (r = 0.52, p<0.005) and the received radiation dose (r = 0.48, p<0.0001). Several prognostic factors of trismus were also detected with signifcant differences between trismus improving and worsening patients, which were the mean dose of the masticator structures (p = .04), masticator muscles (p = .05), lateral pterygoid muscle (p = .04), parotid gland (p = .01), the product of six month SA scores with the mean masticator muscle dose (p = .04), and twelve month SA scores (p = .01). With the optimum cut-off points as 0.38 for the propensity score, we achieved 100% sensitivity with 93% specificity to predict the prognosis of these trismus patients.

Trismus is a well known complication of head and neck cancer treatment [9], [20]. In a previous study, 35 NPC patients completed RT treatment with trismus with a dental gap less than 2.5 cm being studied by retrospective MRI analysis [6]. Fifty-four percent of them had abnormalities in several structures involved with mastication, without mentioning the correlation between RT doses. Several previous studies reported a direct correlation between RT dose of the masticator muscles and subsequent reductions in the dental gap [21], [22], [23]. Dijkstra et al [5] also reported reduced mouth opening of 18% in patients treated by RT involving the structures of the TMJ and/or pterygoid muscles. Similar conditions were also noted in our OCC patients who received RT or CCRT. In the current study, several abnormalities of the masticator muscles were noted by MRI, suggesting trismus is a multifactorial disease. Additionally, there was a strong correlation between the two year SA score summation with the radiation dose received by individual masticator structures (r = 0.48, p<0.0001). This meant our SA score can reflect the radiation dose received by each individual masticator structures and also implied the radiation-induced fibrosis or injury of OCC patients can be revealed by MRI and quantified by our scoring system.

The SA score based on the serial MRI findings proved to have good correlation (r = 0.52, p<0.005) with the clinical trismus severity, hinting that the severity of trismus could be predicted by imaging. According to the results of the ROC curve, patients experiencing some degree of trismus-related impaired eating could be predicted with an SA score higher than 5.5 (with 64% sensitivity and 61% specificity respectively). This suggests the SA score may serve as an imaging predictor of trismus and may also reflect the degree of underlying pathological changes. Moreover, the SA score gradually decreased along with clinical trismus grade and time sequence, which corresponds to our clinical experiences that most radiation-induced trismus patients after IMRT will gradually improve over time. This implies the SA score can be applied as an indicator of treatment response in trismus during follow up.

Our results also revealed some trismus subsided over time, but some did not. For the patient whose trismus will not improve as time goes by, a more aggressive treatment plan including rehabilitation exercise [24], Botox injection or even surgical intervention [25] should be suggested as early as possible. Therefore, it is critical to find the prognostic factors of trismus after RT to help physicians and patients overcome this sequela. In the current study, the six factors correlated with prognosis were the mean dose of masticator structures, masticator muscles, lateral pterygoid muscle, parotid gland, the product of six month SA scores with mean dose of masticator muscles and the twelve month SA scores. Here, we also found the radiation dose of the parotid glands plays a role in trismus prognosis. This may due to the fact that the anatomical correlation between parotid glands and masticator structures influences the dose distribution in RT plan. According to our model, applying the optimum cut-off point of the propensity score at 0.38, we are able to pick out poor-prognostic trismus patients with 100% sensitivity and 93% specificity, which is better than predicted by radiation dose (sensitivity and specificity around 63%/90%). So it considers more factors and thus has better prediction strength. Therefore, in terms of improving the validity of screening, we recommend using all six of these parameters together rather than any one of them alone to enhance the detection of poor prognosis trismus.

If the doses of massetor or pterygoid muscles are larger than 55 Gy, the incidences of trismus for head and neck patients were as high as 45% [15]. Additionally, there is an increased probability of trismus of 24% with every additional 10 Gy to the pterygoid muscle [5]. Practically, according to the previous report and data revealed here, the constraints of the masticator structures would be better below the cut-off values, emphasizing the lateral pterygoid muscle (42 Gy) and parotid gland (28 Gy). For those cases who cannot achieve this goal, the patients would experience a high risk of developing poor-prognosis trismus and some preventive management should be given. Further, if the product of six month SA scores with the mean dose of masticator muscles or the twelve month SA score itself exceeds the proposed cut-off at the 6^th^ or 12^th^ month follow-up after RT, aggressive treatment or intervention for the trismus should be prescribed to facilitate disease improvement.

There are some limitations to the current study. First, due to the nature of retrospective study, we were unable to match the age and gender in each of our groups. In addition, we did not measure the mean incisor distance of each patient and the trismus severity grading system we used may be affected by other factors. Finally, the patients enrolled in the current study should experience post-RT period for more than 2 years due to the deterioration of radiation-induced trismus occurring within 24 months after RT limited the number of patients. All these factors could possibly cause some bias in our results.

MRI SA score and the radiation dose of masticators muscles are useful in predicting trismus severity per se and its prognosis in OCC patients after RT or CCRT. Though more investigations are mandatory to validate our results, the radiation and imaging parameters may serve as a powerful tool to guide the management of trismus after radiation therapy. In addition, one may prevent the development of poor-prognostic trismus by the constraints suggested here, especially on the lateral pterygoid muscles and also parotid glands. If the radiation dose of specific structures or the SA scores at six and twelve months are higher than the proposed cutoffs, more aggressive treatment for trismus is suggested. Long-term follow-up and large prospective studies are needed to confirm these preliminary findings.
